# Whispering Gallery Mode Resonators for Rapid Label-Free Biosensing in Small Volume Droplets

**DOI:** 10.3390/bios5010118

**Published:** 2015-03-23

**Authors:** Sarah M. Wildgen, Robert C. Dunn

**Affiliations:** Ralph N. Adams Institute for Bioanalytical Chemistry, University of Kansas, 2030 Becker Drive, Lawrence, KS 66047, USA; E-Mail: swildgen@ku.edu

**Keywords:** whispering gallery mode resonators, label-free, small volume, rapid assay, real time analysis, sessile droplets

## Abstract

Rapid biosensing requires fast mass transport of the analyte to the surface of the sensing element. To optimize analysis times, both mass transport in solution and the geometry and size of the sensing element need to be considered. Small dielectric spheres, tens of microns in diameter, can act as label-free biosensors using whispering gallery mode (WGM) resonances. WGM resonances are sensitive to the effective refractive index, which changes upon analyte binding to recognition sites on functionalized resonators. The spherical geometry and tens of microns diameter of these resonators provides an efficient target for sensing while their compact size enables detection in limited volumes. Here, we explore conditions leading to rapid analyte detection using WGM resonators as label-free sensors in 10 μL sample droplets. Droplet evaporation leads to potentially useful convective mixing, but also limits the time over which analysis can be completed. We show that active droplet mixing combined with initial binding rate measurements is required for accurate nanomolar protein quantification within the first minute following injection.

## 1. Introduction

The specific detection of biological markers in complex matrices is important in early disease detection, evaluating response to treatment, and monitoring disease reoccurrence. To reduce the complexity, costs, and time associated with these tests, much work has been devoted to developing biosensing methods that can specifically quantify analytes of interest in biological fluids. Numerous sensing schemes have been developed to translate a specific analyte recognition step into a measureable signal. Label-free approaches capable of multiplexed detection are particularly attractive for these applications since they simplify the chemistry and enable multiple targets to be evaluated [1,2,3,4].

One biosensing approach capable of multiplexed, label-free detection uses light trapped in small resonators to sense analyte binding at the sensor surface [5,6,7]. Whispering gallery mode (WGM) resonators are axially symmetric dielectric structures that are tens of microns in diameter. Light coupled into these resonators is efficiently trapped as interactions with the resonator surface leads to continuous total internal reflection [8,9]. When light traveling around the resonator returns in phase, resonances are observed which are characterized by narrow bandwidths and large quality factors (Q-factors) [8,9,10,11,12]. WGM resonances shift in response to changes in refractive index, which leads to interesting applications in label-free sensing [4,5,6,7,13,14,15,16,17].

Historically, the primary metric driving sensor development has been detection limits. This has led to the advancement of many clever biosensing schemes, which enable detection down to the single molecule or particle level [18,19,20,21,22,23]. Often this is accomplished using nanoengineered sensors where the small size of the sensing element leads to favorable signal-to-noise [21,22,23]. Recently, however, an increasing appreciation for the time required to carry out an analysis, especially at low concentrations, has focused a renewed interest in mass transport aspects of biosensing [24,25,26].

For species at low concentration, diffusion to the sensing element often represents a limiting factor in the time required to carry out analysis. Analyte diffusion can lead to surprisingly long mass transport times in unstirred solutions [24,25,26]. For sensing, these issues can be further exaggerated by the size and geometry of the detection element itself. While nanometric sensors offer many unique advantages for sensing, their small size limits diffusion to the active area. At low picomolar analyte concentrations, for example, diffusion to nanoscale elements on a planar surface can take days, leading to impractically long analysis times [24,25,26].

Recently, single molecule and particle detection have been demonstrated with WGM resonators using plasmonic particles to enhance the signal [18,19,20]. Moving to larger WGM microsphere resonators, moreover, can decrease analysis time through favorable mass transport metrics. Their spherical geometry and tens of microns diameters lend themselves to optimized mass transfer for rapid analysis [24,25,26]. Their compact size also enables accurate analysis in small volumes, enabling multiplexed sensing in sample-limited applications. For the latter, small droplet analysis platforms offer an intriguing approach for reducing the costs, complexity and consumables associated with analysis [27,28,29]. Droplets on a surface undergo complex evaporation processes leading to temperature gradients which produce a natural mixing effect within the liquid [30,31,32,33,34]. This can potentially increase sensor response time through improved mass transport.

Here, we investigate the response time of WGM resonators in small droplet assays. As a model system, 38 μm diameter barium-titanate resonators are functionalized with bovine serum albumin-biotin to detect binding of streptavidin. WGM resonators are loaded into 10 μL droplets of phosphate buffered saline supported on a hydrophobic Teflon AF film. Shifts in WGM resonance with time are used to quantify evaporation of the droplets and define the useful limits over which assays can be performed without significant perturbation due to decreasing volume. Sensor response is compared in both stirred and unstirred droplets with the results showing that active stirring is required to sufficiently shorten response time in order to compete with evaporation. Initial binding rate studies in actively stirred droplets show that nanomolar protein concentrations can be quantified within minutes using the small volume assay.

## 2. Experimental Section

### 2.1. Materials

Streptavidin and 10× phosphate buffered saline (PBS) (27 mM KCl; 15 mM KH_2_PO_4_; 1400 mM NaCl; 81 mM Na_2_HPO_4_) were obtained from MP Biomedicals (Solon, OH, USA). Prior to use, 1× PBS solutions were prepared with 0.05% sodium azide (pH 7.35). Bovine serum albumin-biotin (BSA-biotin) and SuperBlock^TM^ (PBS) Blocking Buffer were acquired from Thermo Scientific (Waltham, MA, USA). Unless otherwise noted, all other materials and reagents were obtained from Fisher Scientific (Hampton, NH, USA) and used without further purification.

### 2.2. Microsphere Functionalization

Barium-titanate microspheres (38 μm diameter, Mo-Sci Corp., Rolla, MO, USA) are plasma cleaned and sonicated in H_2_O_2_ for 15 min. The microspheres are rinsed sequentially in water, ethanol, and toluene before reacted in 10% (3-aminopropyl)triethoxysilane (APTES)/toluene for 2 h. Following silanization, spheres are rinsed in toluene, ethanol, and PBS then further reacted in 10% gluteraldehyde/PBS for 1 h. Aldehyde functionalized microspheres are reacted with BSA-biotin for 2 h leading to covalent attachment. To minimize non-specific binding, microspheres are incubated in SuperBlock blocking buffer solution for 15 min. Functionalized microspheres were stored in PBS at 4 °C.

### 2.3. Bioassay Protocol

To fabricate the hydrophobic surfaces, glass microscope slides were spin coated with a thin layer of Teflon^®^ AF amorphous fluoropolymer (400s2-100-1, DuPont, Wilmington, DE, USA). Approximately 1.5 μL of Teflon AF was pipetted onto the clean glass slide and immediately spun at approximately 4000 rpm for ~15 s. Coated slides were placed in a 60 °C oven for 15 min to remove residual solvent and stored at room temperature prior to use.

For refractive index measurements, the appropriate amount of 18 MΩcm water (10 or 100 μL) was pipetted onto unfunctionalized barium-titanate microspheres resting on the Teflon AF coated glass slides. Appropriate volumes (1 μL or 10 μL) of 1.7 M NaCl stock solution were quickly pipetted into the droplet to increase the refractive index by 0.002 refractive index units (RIU). For experiments involving stirred droplets, a 125 μm diameter glass rod was positioned in the droplet prior to the NaCl spikes and rotated at ~5000 rpm.

For protein assays, functionalized BSA-biotin microspheres were placed on a Teflon AF coated glass slide and 10 μL of PBS was carefully pipetted onto them. 1 μL injections of streptavidin/PBS stock solutions (0–7.6 μM) were delivered into the droplet using pipette tips coated with Sigmacote^®^ (Sigma-Aldrich, St Louis, MO, USA) to reduce wetting of the pipette surface.

### 2.4. Experimental Set-Up

**Figure 1:**
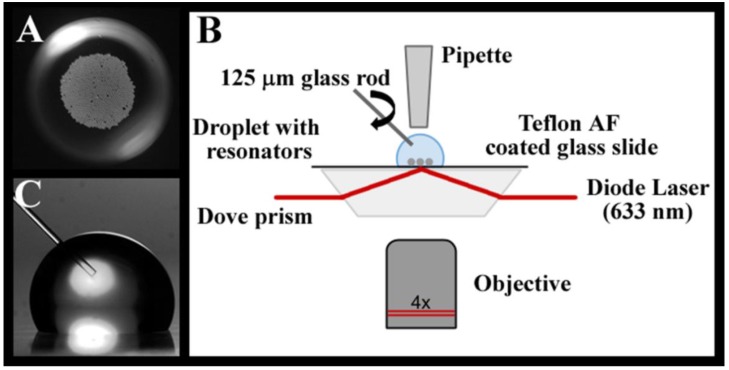
(**A**) Approximately 500 barium-titanate (38 μm diameter) microspheres within a 10 μL PBS droplet on a hydrophobic Teflon AF coated glass surface. (**B**) Schematic of whispering gallery mode (WGM) droplet assay. Light from a tunable diode laser is directed into a Dove prism for evanescent wave coupling into 38 μm barium-titanate microspheres. Evanescently scattered light from the resonators is imaged from below (4× objective, 0.10 NA) and detected on an avalanche photodiode (APD). (**C**) For active mixing experiments, a 125 μm diameter glass rod is rotated at ~5000 rpm in the 10 μL PBS droplet.

The Teflon AF coated glass slide with PBS droplet is placed on a Dove prism coated with high index immersion oil as illustrated in Figure 1B. Excitation light from a tunable diode laser (New Focus Vortex II TLB-6900) centered at wavelength 633 nm is coupled into the prism. The Dove prism refracts the light towards the sample interface at an angle leading to total internal reflection at the interface. The associated evanescent field is used to excite whispering gallery modes in the barium-titanate microspheres.

To measure WGM spectra, the evanescently scattered light from the resonator is collected from below using an inverted optical microscope (Zeiss Axiovert 100 TV) equipped with a 4× objective (Olympus PlanN, 0.10 NA). The scattered light is detected on an avalanche photodiode (APD) (SPCM-200, EG&G). A computer controlled acquisition system synchronizes the wavelength scanning of the laser and data acquisition to continuously record spectra with time. All experiments were carried out at 21 °C ± 1 °C and relative humidities between 10% and 18%.

## 3. Results and Discussion

### 3.1. WGM Response in Small Volumes

Because of their compact size, microsphere WGM resonators are well suited for small volume analysis. Figure 1A, for example, shows approximately 500 resonators (38 μm diameter) loaded into a 10 μL droplet of PBS on a hydrophobic surface. In theory, each resonator in the droplet acts as an individual assay, which enables large signal averaging and/or multiplexing capabilities even in these modest volumes. WGM resonances in the resonators are excited using the evanescent field created at the substrate interface through total internal reflection of the excitation light in a Dove prism, as shown in Figure 1B. To measure the WGM spectrum, evanescently scattered light from a WGM resonator is collected from below with a 4× objective (Olympus PlanN, 0.10 NA) as the excitation wavelength of the laser is tuned. The scattered light can be imaged onto a CCD camera (Photometrics, CoolSnap K4) for multiple resonator analysis or focused onto a single element detector (APD, SPCM-200, EG&G) for rapid analysis of a single resonator. A micropipette positioned above the droplet is used to quickly inject standards into the droplet. Sensor response in unstirred and stirred droplets is compared. For actively mixed droplets, a 125 μm diameter glass rod is positioned in the droplet as shown in Figure 1C and rotated at ~5000 rpm.

Analysis in supported droplets exposed to air is complicated by evaporation. While droplet evaporation can be exploited as a means of concentrating analytes and optimizing detection limits, it more often hinders analysis by limiting the time available [35]. Evaporation of supported sessile droplets is a complicated process that depends on environmental factors such as relative humidity and temperature, as well as, the nature of the interactions between the droplet and the substrate surface [36,37,38,39,40,41]. Here we study aqueous droplets supported on a glass substrate that has been spin coated with the hydrophobic polymer Teflon AF. This leads to droplets with an approximate contact angle of 115° [32]. As others have shown, a high contact angle leads to a linear decrease in droplet volume with time, which can be characterized by measuring the changes in the droplet height [31,32,37].

**Figure 2:**
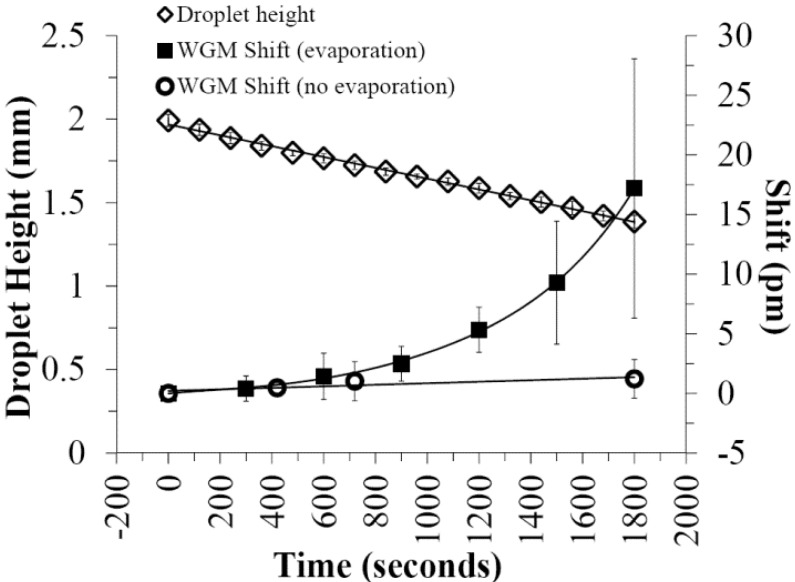
Evaporation of 10 μL PBS droplets on a Teflon AF coated surface (115° contact angle) leads to the expected linear decrease (*R*^2^ = 0.997) in droplet height with time (open diamonds, *N* = 3). The evaporating 10 μL PBS droplet red-shifts the WGM resonance (squares, *N* = 10) of embedded resonators as the decreasing volume concentrates the buffer ions. As shown, evaporation negligibly affects the WGM resonance (≤2 pm shift in first 10 min) early in the evaporative process. Control experiments using 10 μL PBS droplets covered in silicon oil (open circles, *N* = 2) shows the stability of the WGM resonance in the absence of evaporation. Error bars represent inter assay variability.

Figure 2 shows a plot of droplet height with time showing the linear dependence expected. Under the conditions tested, a 10 μL droplet on the hydrophobic surface will typically evaporate in 50–60 min. Also plotted in Figure 2 is the shift in WGM resonance from WGM resonators in an evaporating 10 μL PBS droplet. As the droplet volume decreases, the concentration of ions in the PBS droplet increases, changing the refractive index of the solution and shifting the WGM resonance. In a control study with 10 μL PBS droplets covered in silicon oil to prevent evaporation, negligible shifts in the WGM resonance were observed on this time scale (Figure 2). For droplets in air, the refractive index shifts can complicate specific biosensing at the WGM resonator. However, as shown in Figure 2, the shifts are minimal (≤2 pm) within the first 10 min, thus providing a window over which WGM bioassays can be carried out with negligible effects from background refractive index changes. The goal here, then, is to explore conditions under which analytes can be quantified in small droplet assays before evaporation significantly affects the WGM resonance or droplet volume.

**Figure 3:**
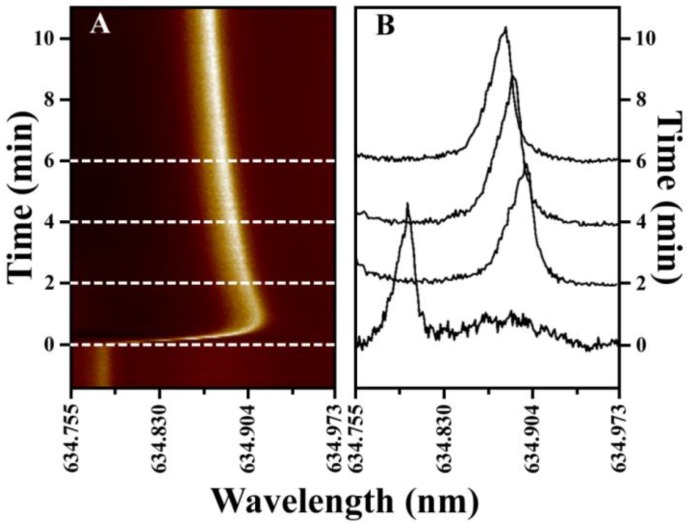
(**A**) Waterfall plot showing the time evolution in the WGM resonance of a 38 μm diameter barium-titanate resonator in a 100 μL water droplet. At time zero, a 10 μL spike of saline solution (ΔRIU = +0.002) is quickly injected into the droplet, elevating the refractive index and red-shifting the WGM resonance. The WGM resonance relaxes towards a new equilibrium position as convective mixing in the sessile droplet equilibrates the solution. (**B**) Selective line cuts from the waterfall plot show the time evolution in the WGM resonance following saline injection. The measured Q-factor is 3.0 × 10^4^.

To minimize the effects of evaporation, initial binding rate measurements are used to quantify protein concentration. These measurements are rapid since they do not require the establishment of equilibrium and offer additional advantages for microsphere WGM assays, where resonator Q-factor and other metrics difficult to control can lead to variations in resonator sensitivity. First, however, WGM resonator response in small volume droplets must be characterized and optimized. To characterize sensor response, spectra are continuously recorded with time as shown in Figure 3. The waterfall plot in Figure 3A shows the response from a 38 μm resonator in a 100 μL droplet of water following a rapid injection of salt solution to elevate the refractive index. Figure 3B highlights selected line cuts from the waterfall plot showing the evolution in the WGM spectra with time. The WGM resonance has a measured Q-factor of 3.0 × 10^4^.

**Figure 4:**
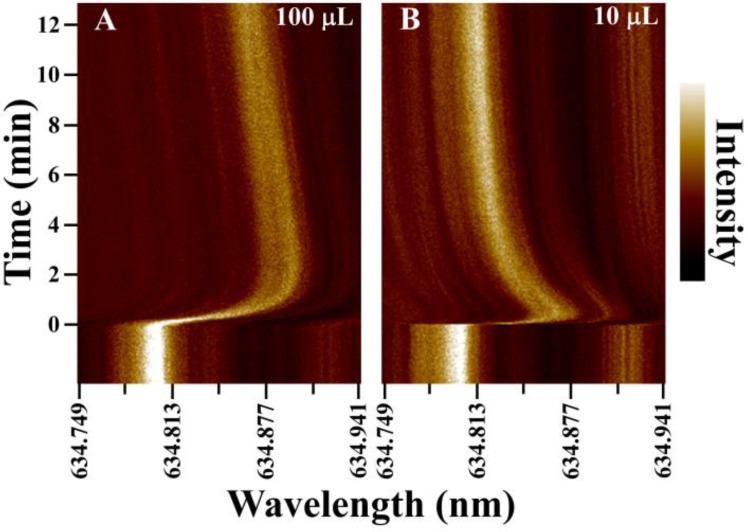
Comparison of the WGM response in unstirred 100 μL (**A**) and 10 μL (**B**) water droplets following a saline injection to raise the refractive index 0.002 RIU. The same WGM resonator was used in both experiments. Convective mixing in the droplets equilibrates the solution in minutes, with the smaller droplet requiring less time.

To characterize resonator response in small sessile droplets, Figure 4 compares representative WGM shifts with time for two different droplet volumes. Figure 4A,B compare the response of the same WGM resonator in a 100 μL and a 10 μL droplet, respectively, upon injection of salt solution to increase droplet refractive index 0.002 RIU. Both time traces show an initial maximum shift in the WGM resonance as the injected high index solution interacts with the resonator. These shifts relax towards an equilibrium value as convective currents in the sessile droplets mix the solution.

Evaporation leads to convective mixing in droplets, which offers an intriguing route for increasing analyte mass transfer to the sensing element. Counter rotating vortices are observed in evaporating droplets that create distinct flow patterns within the drop [30,31,32,33,34]. For multicomponent droplets, such as those used in assays, evaporative associated density gradients also develop within the droplet and can contribute to increasing internal flow velocities [32,33]. These flow velocities can be orders of magnitude greater than diffusive flow. However, as illustrated in Figure 4, even with these convective flows, complete mixing takes minutes as measured by refractive index changes at the WGM resonator. Even for small droplets (10 μL), the signal in Figure 4B requires approximately 10–12 min to relax to its new equilibrium value following the refractive index spike. When compared with the evaporation data in Figure 2, these mixing rates are not sufficiently fast to avoid complications from evaporation. This suggests that convective mixing in sessile droplets is not adequate and active mixing is required to achieve sufficient mass transfer rates. 

**Figure 5:**
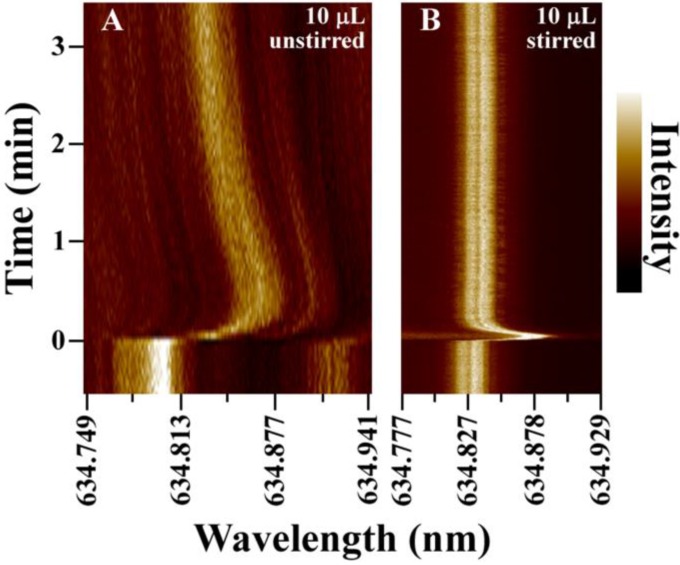
Comparison of the WGM response in unstirred (**A**) and stirred (**B**) 10 μL nanopure water droplets following injection of saline to increase the refractive index 0.002 RIU. For measurements on actively mixed droplets, a 125 μm diameter glass rod was inserted into the drop and rotated at ~5000 rpm as shown in Figure 1C. With stirring, the WGM resonance establishes the new equilibrium value within seconds of the injection.

For active mixing, a 125 μm diameter stirring rod was positioned in the droplet as shown in Figure 1C. The rod was rotated at ~5000 rpm to vigorously mix the small droplet during the NaCl solution spike. The results are summarized in Figure 5, which compares the response time in an unstirred 10 μL droplet with that of a stirred 10 μL droplet using the rotating rod. A significant improvement in sensor response time is measured in the stirred droplet. Both traces show an initial large shift in WGM resonance as the refractive index spike is delivered into the droplet. For the stirred droplet, however, the signal quickly relaxes to the equilibrium value in seconds as efficient mixing equilibrates the droplet following injection.

**Figure 6:**
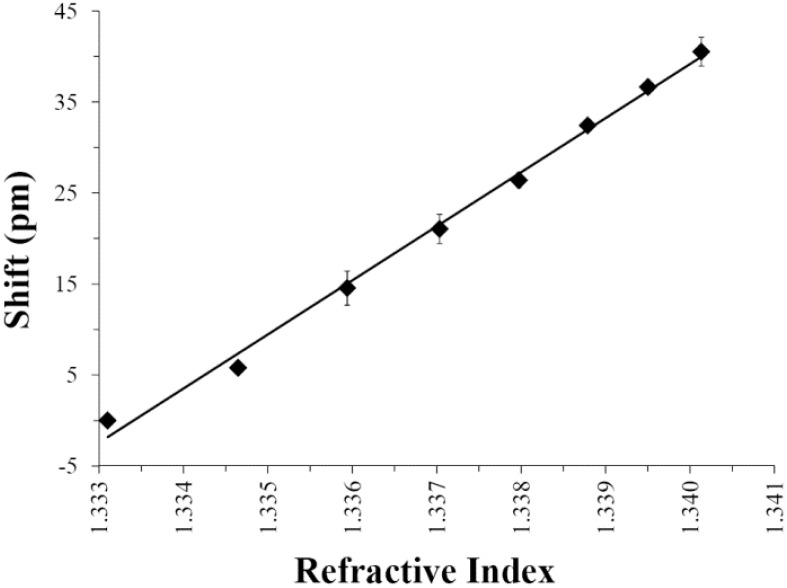
Refractive index calibration plot for an unmodified 38 μm WGM resonator in a stirred 10 μL water droplet. Injections of 1.7 M NaCl stock solution were used to increase refractive index in the drop. The calibration plot indicates a linear response (*R*^2^ = 0.995) and measured limit of detection of 6.2 × 10^−4^ RIU. Error bars represent intra assay variability (*N* = 3).

To validate that active mixing enables reliable sensor response in small droplets, Figure 6 shows a representative refractive index calibration plot. For these measurements, a 10 μL droplet of nanopure water loaded with 38 μm barium-titanate resonators was supported on a hydrophobic Teflon AF surface. While stirring, 1 μL additions of 1.7 M NaCl solutions were spiked into the droplet to increase the refractive index and WGM resonance shifts were tracked in real time. Figure 6 indicates the linear trend (*R*^2^ = 0.995) between the measured resonant wavelength (taken 1 min after each injection) and droplet refractive index. Importantly, the entire calibration was completed in less than 10 min with a measured limit of detection of 6.2 × 10^−4^ RIU.

### 3.2. Small Volume Protein Binding Assay

**Figure 7:**
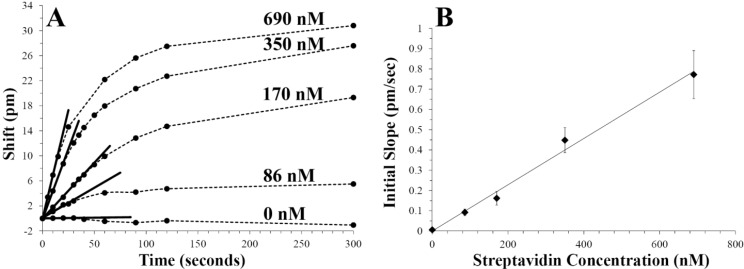
(**A**) For rapid protein quantification, biotin-streptavidin binding rates are measured as a function of streptavidin concentration. Each plot represents multiple, separate assays carried out in a 10 μL PBS droplet using rapid stirring. 1 μL injections of streptavidin/PBS solutions (0–7.6 μM) were quickly made in 10 μL PBS droplets containing BSA-biotin functionalized 38 μm spheres. Continuous measurement of WGM resonant wavelength shifts enables initial binding rates to be accurately quantified as a function of streptavidin concentration. Control experiments using unfunctionalized resonators showed no shifts following injections with 1 μL of 1.9 μM streptavidin/PBS (data not shown). (**B**) Initial binding rates measured from (**A**) are plotted *versus* streptavidin concentration showing the linear trend (*R*^2^ = 0.991). Initial binding rates are determined within the first minute following protein injection, enabling rapid quantification in a small volume. Error bars represent inter assay variability at each streptavidin concentration (*N* = 3).

Having established a rapid refractive index sensing scheme for 10 μL droplets, initial binding rate studies were carried out using biotin-streptavidin as a model system. For each assay, 38 μm diameter resonators functionalized with biotin were immersed in a 10 μL droplet of PBS. The droplets were mixed with the rotating rod and 1 μL spikes of streptavidin/PBS stock solutions (0–7.6 μM) were quickly injected into the droplet. Figure 7A compares the time evolution of WGM resonances as a function of streptavidin concentration. Each trace in Figure 7A represents multiple, separate assays using different functionalized WGM resonators to compare response times with streptavidin concentration. As expected, the initial rate of change and final equilibrium shift in the WGM resonance increases with increasing concentration of streptavidin added.

With rapid stirring, Figure 7A shows that WGM shifts reflecting initial binding rates can be quantified in the first 60 s following injection. Control experiments using unfunctionalized resonators, moreover, show that non-specific binding or droplet refractive index changes from evaporation do not contribute to the measured signals. Linear fits to the initial slopes in the binding data are shown in Figure 7A and the extracted slopes are plotted as a function of added streptavidin in Figure 7B. The linear response with protein concentration (*R*^2^ = 0.991) illustrates the utility of this quantitative approach for quickly measuring nanomolar protein concentrations in small volumes.

Biosensing in small volumes opens new opportunities in sample-limited applications. The small volumes combined with active mixing are shown to lead to very efficient mass transport of analytes to the sensing surface. This, combined with the favorable geometry and size of WGM resonators, results in rapid analysis times for nanomolar analyte concentrations. The biotin-streptavidin system studied here offers especially favorable binding characteristics. Capture agents and targets with lower binding affinities or rates will likely require longer analysis times. Lower analyte concentrations will also require longer analysis times which will eventually lead to complications arising from droplet evaporation. For these applications, more involved approaches to limit or eliminate evaporation using either well-controlled environmental conditions or protective oil layers can be implemented.

## 4. Conclusions

Small volume droplet assays utilizing WGM resonators are demonstrated as a promising label-free sensing platform. The geometry and size of WGM microspheres make them well suited for rapid mass transport and small volume analysis. The results presented here show that convective mixing in sessile droplets is not sufficiently rapid to enable nanomolar protein quantification on a time scale competitive with evaporation. Without active mixing, effects of evaporation on droplet volume, ion concentration, and protein concentration can all complicate protein quantitation using WGM resonators. However, active mixing of the small droplets using a small, spinning glass rod is shown to result in rapid mass transfer and efficient sensing on short time scales. With active mixing, sensor response times are reduced to seconds, which enables nanomolar protein quantitation using initial binding rates within the first minute following injection.
